# Retraction: Automated brain tumor diagnostics: Empowering neuro-oncology with deep learning-based MRI image analysis

**DOI:** 10.1371/journal.pone.0353921

**Published:** 2026-07-16

**Authors:** The PLOS One Editors

The *PLOS One* Editors retract this article [1] due to concerns about:

- Non-compliance with PLOS policy on Authorship.
- Text overlap with a previously published article [2] which does not share authors with [1].
- Unreliable peer review.
- Citation of Reference 15 which was retracted before [1] was published.

PSMB, SKM, and BDS did not agree with the retraction. SG, HR, and MAS either did not respond directly or could not be reached.
